# Burden of Helicobacter pylori in Duodenal Ulcer Disease: Evidence From a Tertiary Hospital in Chennai, India

**DOI:** 10.7759/cureus.90573

**Published:** 2025-08-20

**Authors:** Arunima Das, Srinivasan C, Jaykanthan N, Velvizhi Ravi, Suganya P

**Affiliations:** 1 General Surgery, Sree Balaji Medical College and Hospital, Chennai, IND

**Keywords:** antibiotic resistance, duodenal ulcer, eradication therapy, helicobacter pylori, prevalence, risk factors, ugi scopy

## Abstract

Background: Duodenal ulcers are strongly linked to a Gram-negative, urease-producing bacterium *Helicobacter pylori *(*H. pylori*) infection. Despite significant global research, there is a notable gap in regional studies detailing its prevalence and clinical patterns in South India, particularly Chennai. With growing antibiotic resistance and the influence of lifestyle factors, understanding the epidemiology of *H. pylori *infection becomes crucial for managing the disease successfully.

Aim: To determine the prevalence of *H. pylori* infection among patients diagnosed with duodenal ulcers at a tertiary care hospital in Chennai, India.

Methods: A cross-sectional study was performed over one year at Sree Balaji Medical College and Hospital in Chennai. It included 112 patients diagnosed with duodenal ulcers via endoscopy. *H. pylori* detection involved multiple methods: biopsies obtained during upper gastrointestinal (UGI) endoscopy were subjected to histopathology and rapid urea test (RUT), while patients also underwent stool antigen test and urea breath test. Data encompassing clinical characteristics, risk factors, hygiene practices, and treatment protocols were collected via structured interviews and analyzed with Statistical Product and Service Solutions (SPSS, version 25.0; IBM SPSS Statistics for Windows, Armonk, NY).

Results: Overall prevalence of *H. pylori *among duodenal ulcer patients was 78 patients (69.6%). Infection rates were the highest in the 31-50 year age group and were slightly higher among females, which was 36 in total (72%), than the 42 males (67.7%). Significant associations were identified in *H. pylori* infection and risk factors such as smoking, alcohol use, high-salt diet, nonsteroidal anti-inflammatory drug (NSAID) use, poor hygiene practices, and a family history of peptic ulcer disease. Histopathology was the most accurate diagnostic method, showing 100% sensitivity and specificity. Quadruple therapy resulted in a higher eradication rate, which was for 26 patients out of 28 patients (92.9%). A lower six-month ulcer recurrence in two patients (7.1%) compared to triple therapy (76.0% eradication; 24.0% recurrence) was significantly evident. Adverse effects were more common with quadruple therapy but were manageable. Complications such as gastrointestinal bleeding and longer hospital stays were considerably more prevalent among *H. pylori*-positive patients.

Conclusion: This research highlights the high burden of *H. pylori* among duodenal ulcer patients in Chennai. Regional risk factors, including diet, hygiene, and medication use, contribute significantly to infection prevalence. Quadruple therapy showed superior efficacy in the eradication and prevention of recurrence. These findings support the need for routine screening, region-specific treatment protocols, and targeted public health interventions to control *H. pylori*-related gastroduodenal disease.

## Introduction

A unique characteristic of *Helicobacter pylori* is its capability to colonize gastric epithelium and persist for decades. This Gram-negative bacterium is spiral-shaped and thrives in low oxygen conditions. Since Barry Marshall and Robin Warren's significant discovery in 1984, *H. pylori *has been definitively linked to peptic ulcer disease (PUD), gastric adenocarcinoma, chronic gastritis, and mucosa-associated lymphoid tissue (MALT) lymphoma [1]. Their Nobel Prize-winning work fundamentally changed the pathogenesis of ulcer, shifting the focus from stress and diet to microbial etiology.

Biological features and pathogenesis

The capability of *H. pylori* to thrive in a highly acidic stomach environment has been facilitated by urease enzyme production, which hydrolyzes urea into carbon dioxide and ammonia. This process neutralizes gastric acid locally, permitting bacteria to establish a niche beneath the mucus layer [2]. Its flagella enable movement, and adhesins help it stick to epithelial cells. Furthermore, virulence features, namely, vacuolating cytotoxin A (Vac A) and cytotoxin-associated gene A (Cag A), affect cellular functions, trigger inflammation, and alter immune responses. [3] These mechanisms collectively contribute to gastric mucosal damage, increased acid secretion, impaired mucosal defenses, and progression to ulceration or neoplasia.

Global prevalence and variation

About 4.4 billion people worldwide suffer from *H. pylori*, one of the most common chronic illnesses. Prevalence is disproportionately high in low- and middle-income nations, exceeding 70% areas of Africa, Asia, and Latin America, compared to 30-50% in most industrialized nations [4]. The disparity is largely attributed to poor sanitation, unsafe drinking water, household overcrowding, and limited healthcare access [5]. Transmission typically occurs through oral-oral or fecal-oral routes, often within families during childhood. However, despite the widespread nature of infection, only 10-20% of those infected develop symptomatic disease, suggesting the influence of host genetics, environmental exposures, and bacterial strain variation [6].

Burden in India and the regional context

Within India, the reported prevalence of *H. pylori *infection ranges from 60% and 80%, with even higher rates recorded in children and lower socioeconomic populations [7]. Several studies from North and South India have consistently demonstrated a high correlation between *H. pylori *infection and duodenal ulcer disease. Instead, limited information exists specific to Chennai, one of India’s most densely populated and medically advanced cities. With its diverse patient demographics and significant burden of gastrointestinal (GI) diseases, there is a critical need to study *H. pylori *prevalence in this local context to inform clinical practice and public health policy.

Duodenal ulcers and clinical manifestations

*H. pylori *plays a causative role in up to 85-90% of duodenal ulcer cases, making it one of the most well-defined infectious causes of chronic GI disease [8]. Duodenal ulcers typically present with epigastric pain, postprandial discomfort, nausea, and, in severe cases, melena, hematemesis, or perforation. The underlying mechanism involves increased gastric acid production and disruption of duodenal bicarbonate secretion, facilitated by chronic gastritis initiated by *H. pylori*. Over time, persistent infection leads to mucosal atrophy, dysplasia, intestinal metaplasia, and ultimately gastric cancer in a subset of patients [9].

Complications and long-term risks

Whether left untreated, *H. pylori*-associated ulcers could result in significant complications such as ulcer perforation, upper GI bleeding, malignant transformation, and gastric outlet obstruction. The World Health Organization (WHO) has designated *H. pylori* a Class I carcinogen, based on its strong epidemiological connection with gastric adenocarcinoma [10]. A large Danish cohort study explained that patients with duodenal ulcers had an ultimately higher risk of developing GI cancers over long-term follow-up, reinforcing the need for early detection and eradication [11].

Diagnostics: evolving modalities

Diagnosis of *H. pylori* has evolved considerably, transitioning from invasive biopsy procedures to accurate non-invasive approaches. While histology via rapid urease tests (RUT), endoscopic biopsy, and culture are still considered the gold standard for those undergoing upper GI endoscopy, non-invasive techniques such as stool antigen test (SAT) and urea breath test (UBT) are now widely utilized for screening and post treatment verification given their excellent sensitivity and specificity [12]. In settings such as Chennai, where endoscopy is often performed for persistent dyspepsia or ulcer-related symptoms, biopsy-based diagnostics provide an opportunity for accurate diagnosis and real-time histopathological assessment.

Treatment and rising resistance

The standard first-line treatment involves triple therapy (PPI + amoxicillin + clarithromycin). However, clarithromycin resistance has emerged as a major hurdle, reducing eradication rates to below 70% in some regions [13]. This has led to the adoption of bismuth-based quadruple therapy, which has shown superior efficacy. Such a situation is further complicated by rising dual and multi-drug resistance, often due to prior antibiotic exposure, self-medication, and lack of antimicrobial stewardship. These trends emphasize the requirement for region-specific treatment regimens, on the basis of local resistance patterns, and the possible role of antimicrobial susceptibility testing (AST) [14].

Global and national trends in *H. pylori a*mong ulcer patients

In Vietnam, a study found that 80% of symptomatic children undergoing endoscopy were *H. pylori*-positive, and 19% had peptic ulcers [15]. In Lebanon, *H. pylori *prevalence was 52.4%, with significantly higher rates among patients with prior ulcer history [16]. In the United States, long-term surveillance data showed a decline in *H. pylori*-positive duodenal ulcers from 25%-21% over a decade, attributed to improved hygiene and widespread eradication therapy [15]. In India, while multiple studies confirm the high prevalence of *H. pylori *among ulcer patients, the lack of updated hospital-based data from Chennai poses a challenge in understanding local disease burden and resistance patterns. Given the city's healthcare infrastructure and large patient population, focused epidemiological studies are warranted.

High burden of *H. pylori-a*ssociated duodenal ulcers

With *H. pylori *causing the majority of duodenal ulcers globally, identifying its prevalence in Chennai's tertiary care hospitals will provide essential insights for early diagnosis, empirical therapy, and patient education.

Antibiotic resistance and treatment challenges

The decline in efficacy of standard triple therapy due to rising metronidazole and clarithromycin resistance underlines the importance of region-specific data to guide treatment choices and improve eradication outcomes.

Preventing complications and healthcare costs

Timely detection and eradication of *H. pylori *decreases the risk of ulcer-related complications such as perforation, bleeding, and cancer, ultimately lowering healthcare costs and morbidity rates.

Public health significance

Given the high prevalence of *H. pylori *infection in India, effective interventions in public health are necessary to reduce its impact. Screening programs, improved sanitation, and targeted eradication therapies can significantly lower infection rates. This study will provide crucial data that can aid in the formulation of public health policies intended to reduce *H. pylori*-related morbidity. This current investigation seeks to determine the prevalence of *H. pylori* among duodenal ulcer patients in a tertiary hospital in Chennai. By identifying the burden of infection in such a specific patient population, the study will contribute to the development of better therapeutic, diagnostic, and preventive strategies. Given the rising antibiotic resistance and the high incidence of duodenal ulcers in India, research of this nature is imperative for improving clinical outcomes and shaping public health initiatives. This study is to determine the prevalence of *H. pylori *infection among patients diagnosed with duodenal ulcers in a tertiary care hospital in Chennai.

## Materials and methods

Study design

This research is a hospital-based cross-sectional study designed to estimate the prevalence of *H. pylori* infection among patients diagnosed with duodenal ulcers.

Study period

The study was conducted for one year at Sree Balaji Medical College and Hospital (SBMCH), Chennai.

Study population

The study population consisted of patients diagnosed with duodenal ulcers who tested positive for *H. pylori *infection. These patients were identified through endoscopic examination and histopathological confirmation.

The inclusion criteria were patients above 12 years of age presenting with dyspepsia or symptoms as melena, weight loss, and anemia and patients who submitted written informed consent for being a participant in the study.

The exclusion criteria were patients treated for *H. pylori *infection previously, patients who had taken proton pump inhibitor (PPIs) or antibiotics within the past four weeks prior to endoscopy, and patients who refused consent to participate in the study.

Sampling technique

The study employed a consecutive sampling method, meaning that all eligible patients presenting during the research period were involved until the required sample size had been met.

Sample size calculation

The single population proportion formula below was utilized to determine the sample size:

(n = ((Za/2)2 * P (1 − P))/d2)

where Zα/2 = 1.96 (95% confidence level), P = 3.5% (assumed prevalence of surgical site infection based on a reference study), and d = 5% (margin of error). The calculation indicated that a sample size of 52 patients was necessary. The ultimate total sample size was determined at 112 patients, taking into account a 10% non-response rate. The reference for sample size calculation is found in the study by Ebogo Titus et al. [17].

Data collection

A structured questionnaire has been utilized to collect data from patients, including demographic details (age, gender, marital status, occupation, and education); clinical presentation (symptoms such as pain, nausea, vomiting, and bleeding); endoscopic findings (presence of ulcer, ulcer classification using the Forrest classification system); complications (perforation, hemorrhage, and obstruction); treatment history (use of PPIs, antibiotics); and dietary and environmental risk factors (smoking, alcohol, and hygiene practices).

## Results

The study included 112 participants, given a distribution that is almost equal, with 62 males (51.7%) and 50 females (48.3%). The highest number of cases was obtained in the 41-50 years age group (27 patients, 22.5%), followed by the 31-40 years age group (24 patients, 20.0%). The lowest number of cases was found in adolescents (five patients, 4.2%), suggesting that *H. pylori *infection and duodenal ulcers are more prevalent in middle-aged or older individuals (Table 1).

**Table 1:** Age and gender distribution of the study participants

Age Group (in Years)	Male (n=62, %)	Female (n=50, %)	Total (n=112, %)
12-20 years	3 (5.0%)	2 (3.3%)	5 (4.2%)
21-30 years	8 (13.3%)	5 (8.3%)	13 (10.8%)
31-40 years	14 (23.3%)	10 (16.7%)	24 (20%)
41-50 years	15 (25%)	12 (20%)	27 (22.5%)
51-60 years	12 (20%)	10 (16.7%)	22 (18.3%)
>60 years	10 (16.7%)	11 (18.3%)	21 (17.5%)
Total	62 (51.7%)	50 (48.3%)	112 (100%)

Only 9.8% of participants were from high-income families, with the majority (54 patients, 48.2%) coming from middle-income backgrounds and the low-income group (47 patients, 42.0%) coming in second. The findings suggest a possible association between lower socioeconomic status and *H. pylori *infection, probably because of poor sanitation, overcrowding, and dietary factors (Table 2).

**Table 2:** Socioeconomic status of the participants

Socioeconomic Status	Male (n=62, %)	Female (n=50, %)	Total (n=112, %)
Low Income (< ₹15,000/month)	25 (40.3%)	22 (44.0%)	47 (42.0%)
Middle Income (₹15,000-₹50,000/month)	30 (48.4%)	24 (48.0%)	54 (48.2%)
High Income (> ₹50,000/month)	7 (11.3%)	4 (8.0%)	11 (9.8%)
Total	62 (100%)	50 (100%)	112 (100%)

Among *H. pylori*-positive patients, the common symptom was epigastric pain in 65 patients (83.3%), followed by nausea/vomiting in 50 patients (64.1%). Patients with *H. pylori *were twice possible to have melena in 22 patients (28.2%), and those without the infection were five patients (14.7%), explaining a stronger association between *H. pylori *and GI bleeding (Table 3).

**Table 3:** Clinical symptoms and duration of illness in H. pylori-positive and H. pylori-negative patients

Symptom	*Helicobacter pylori*-positive (n=78, %)	*Helicobacter pylori*-negative (n=34, %)	Total (n=112, %)
Epigastric Pain	65 (83.3%)	22 (64.7%)	87 (77.7%)
Nausea/Vomiting	50 (64.1%)	15 (44.1%)	65 (58.0%)
Bloating	40 (51.3%)	10 (29.4%)	50 (44.6%)
Melena (Black Stools)	22 (28.2%)	5 (14.7%)	27 (24.1%)
Weight Loss	35 (44.9%)	10 (29.4%)	45 (40.2%)

Overall, *H. pylori *prevalence among the population was 78 patients (69.6%), suggesting that patients with duodenal ulcers have a high infection burden (Table 4).

**Table 4:** Overall prevalence of H. pylori in the study population

*Helicobacter pylori*-positive	*Helicobacter pylori*-negative	Total
78 (69.6%)	34 (30.4%)	112

The highest prevalence of *H. pylori *has been observed in the 31-50 years age group (38 patients, 74-75%), followed by the 51-60 years age group (16 patients, 68.2%). The lowest prevalence was observed in younger participants (12-20 years, 40.0%), indicating an age-related rise in infection rates (Table 5).

**Table 5:** Prevalence of H. pylori by age group

Age Group (in Years)	*Helicobacter pylori*-positive (n=78, %)	*Helicobacter pylori*-negative (n=34, %)	Total (n=112,%)
12-20 years	2 (40.0%)	3 (60.0%)	5 (4.2%)
21-30 years	9 (69.2%)	4 (30.8%)	13 (10.8%)
31-40 years	18 (75.0%)	6 (25.0%)	24 (20.0%)
41-50 years	20 (74.1%)	7 (25.9%)	27 (22.5%)
51-60 years	15 (68.2%)	7 (31.8%)	22 (18.3%)
>60 years	14 (66.7%)	7 (33.3%)	21 (17.5%)

Females had a slightly greater prevalence of *H. pylori *(36) (72.0%) than males (42) (67.7%), although the difference was statistically not significant (Table 6).

**Table 6:** Prevalence of H. pylori by gender

Gender	*Helicobacter pylori*-positive (n=78, %)	*Helicobacter pylori*-negative (n=34, %)	Total (n=112,%)
Male	42 (67.7%)	20 (32.3%)	62 (55.4%)
Female	36 (72.0%)	14 (28.0%)	50 (44.6%)

*H. pylori *was more prevalent in 58 duodenal ulcer patients (78.4%) as compared to 20 gastric ulcer patients (52.6%), supporting its strong association with duodenal ulcers (Table 7). 

**Table 7:** Prevalence of H. pylori in duodenal ulcer vs. gastric ulcer patients

Ulcer Type	*Helicobacter pylori*-positive (n=78, %)	*Helicobacter pylori*-negative (n=34, %)	Total (n=112,%)
Duodenal Ulcer	58 (78.4%)	16 (21.6%)	74 (66.1%)
Gastric Ulcer	20 (52.6%)	18 (47.4%)	38 (33.9%)

Thirty-five patients with *H. pylori* were prone to smoke (44.9%) than the nine patients without the infection (26.5%), which may indicate an association between smoking and *H. pylori *infection. Similarly, a higher proportion of 30 *H. pylori*-positive individuals reported alcohol consumption (38.5%) compared to eight *H. pylori*-negative individuals (23.5%), indicating a potential link between alcohol intake and increased susceptibility to infection (Table 8).

**Table 8:** Association of H. pylori with smoking and alcohol consumption

Risk Factor	*Helicobacter pylori*-positive (n=78, %)	*Helicobacter pylori*-negative (n=34, %)	Total (n=112,%)
Smokers	35 (44.9%)	9 (26.5%)	44 (39.3%)
Non Smokers	43 (55.1%)	25 (73.5%)	68 (60.7%)
Alcohol Consumers	30 (38.5%)	8 (23.5%)	38 (33.9%)
Alcohol Non-consumers	48 (61.5%)	26 (76.5%)	74 (66.1%)

Fifty (64.1%) *H. pylori*-positive patients following high-salt consumption reported a significant association with *H. pylori *infection. Similarly, a greater proportion of infected patients reported regular intake of spicy and processed foods, suggesting that dietary habits may influence *H. pylori *colonization and persistence (Table 9).

**Table 9:** Dietary habits and H. pylori infection

Dietary Habit	*Helicobacter pylori*-positive (n=78, %)	*Helicobacter pylori*-negative (n=34, %)	Total (n=112, %)
High Salt Diet	50 (64.1%)	12 (35.3%)	62 (55.4%)
Low Salt Diet	28 (35.9%)	22 (64.7%)	50 (44.6%)
Frequent Spicy Food Intake	42 (53.8%)	9 (26.5%)	51 (45.5%)
Rare Spicy Food Intake	36 (46.2%)	25 (73.5%)	61 (54.5%)
Processed Food Consumption	48 (61.5%)	10 (29.4%)	58 (51.8%)

Forty (51.3%) *H. pylori*-positive patients used NSAIDs frequently as compared to eight *H. pylori*-negative individuals (23.5%). Results demonstrate that NSAID application and *H. pylori *infection may act synergistically to raise concern related to PUD, highlighting the importance of careful NSAID prescribing in infected individuals (Table 10).

**Table 10:** Association of H. pylori with NSAID use NSAID: nonsteroidal anti-inflammatory drug

NSAID Use	*Helicobacter pylori*-positive (n=78, %)	*Helicobacter pylori*-negative (n=34, %)	Total (n=112, %)
Yes	40 (51.3%)	8 (23.5%)	48 (42.9%)
No	38 (48.7%)	26 (76.5%)	64 (57.1%)

*H. pylori* infection was substantially linked to a family history of PUD, with 41.0% (32) of infected individuals reporting a family history of ulcers as compared to only 17.6% (6) of uninfected individuals. This suggests a possible genetic or household transmission component in *H. pylori *infection (Table 11).

**Table 11:** Association of H. pylori with family history of peptic ulcer disease

Family History	*Helicobacter pylori*-positive (n=78, %)	*Helicobacter pylori*-negative (n=34, %)	Total (n=112, %)
Present	32 (41.0%)	6 (17.6%)	38 (33.9%)
Absent	46 (59.0%)	28 (82.4%)	74 (66.1%)

Poor hygiene practices, including drinking unfiltered water and infrequent handwashing, were more prevalent in *H. pylori*-positive individuals. Utilization of unfiltered water has been nearly twice as common in 45 infected patients (57.7%) compared to eight uninfected individuals (23.5%), indicating that contaminated water sources may be a significant risk factor for transmission (Table 12).

**Table 12:** Association between H. pylori and hygiene practices

Hygiene Practice	*Helicobacter pylori*-positive (n=78, %)	*Helicobacter pylori*-negative (n=34, %)	Total (n=112, %)
Unfiltered Water Use	45 (57.7%)	8 (23.5%)	53 (47.3%)
Filtered Water Use	33 (42.3%)	26 (76.5%)	59 (52.7%)
Infrequent Handwashing	38 (48.7%)	10 (29.4%)	48 (42.9%)
Frequent Handwashing	40 (51.3%)	24 (70.6%)	64 (57.1%)

The high correlation between *H. pylori* infection along with duodenal ulcer disease was further supported by the fact that duodenal ulcers were frequent in patients with *H. pylori* (58, 74.4%) than in those without *H. pylori (*16, 47.1%) (Table 13).

**Table 13:** Endoscopic findings in H. pylori-positive and H. pylori-negative patients

Findings	*Helicobacter pylori*-positive (n=78, %)	*Helicobacter pylori*-negative (n=34, %)	Total (n=112, %)
Duodenal Ulcer	58 (74.4%)	16 (47.1%)	74 (66.1%)
Gastric Ulcer	20 (25.6%)	18 (52.9%)	38 (33.9%)
Erosive Gastritis	30 (38.5%)	8 (23.5%)	38 (33.9%)
Gastric Atrophy	22 (28.2%)	5 (14.7%)	27 (24.1%)

More severe ulcers (Forrest IA and IB) were predominantly seen in *H. pylori-*positive patients, suggesting that *H. pylori *contributes to ulcer bleeding (Table 14).

**Table 14:** Classification of ulcers based on forrest classification

Forrest Classification	*H. pylori*-positive (n=78, %)	*H. pylori*-negative (n=34, %)	Total (n=112, %)
Ia	10 (12.8%)	2 (5.9%)	12 (10.7%)
Ib	8 (10.3%)	1 (2.9%)	9 (8.0%)
II	20 (25.6%)	6 (17.6%)	26 (23.2%)
III	40 (51.3%)	25 (73.5%)	65 (58.1%)

Histopathology revealed chronic gastritis - 65 patients (83.3%) as the most common outcome among individuals infected by *H. pylori*. Gastric atrophy and chronic active gastritis were significantly more frequent in the infected group, suggesting a strong inflammatory response induced by *H. pylori *(Table 15).

**Table 15:** Histopathological diagnosis of gastric biopsy samples

Histopathological Finding	*Helicobacter pylori*-positive (n=78, %)	*Helicobacter pylori*-negative (n=34, %)	Total (n=112, %)
Chronic Gastritis	65 (83.3%)	20 (58.8%)	85 (75.9%)
Chronic Active Gastritis	48 (61.5%)	10 (29.4%)	58 (51.8%)
Gastric Atrophy	20 (25.6%)	4 (11.8%)	24 (21.4%)
Intestinal Metaplasia	10 (12.8%)	2 (5.9%)	12 (10.7%)

Histopathology had the highest detection rate (78 patients, 69.6%), followed closely by the urea breath test (UBT) (75 patients, 67.0%). These results highlight histology as the most sensitive method, although UBT and stool antigen test (SAT) remain highly effective non-invasive tests (Table 16).

**Table 16:** Comparison of diagnostic tests used for H. pylori detection

Test	Positive (n, %)	Negative (n, %)
Rapid Urease Test	70 (62.5%)	42 (37.5%)
Histopathology	78 (69.6%)	34 (30.4%)
Urea Breath Test	75 (67.0%)	37 (33.0%)
Stool Antigen Test	72 (64.3%)	40 (35.7%)

Histology's stance as the gold standard for diagnosis was confirmed by its high sensitivity and specificity. High sensitivity and specificity were demonstrated by the non-invasive UBT and SAT, suggesting their usefulness in clinical practices (Table 17).

**Table 17:** Sensitivity and specificity of diagnostic tests for H. pylori

Test	Sensitivity (%)	Specificity (%)	Positive Predictive Value (%)	Negative Predictive Value (%)
Rapid Urease Test	85.90%	88.20%	95.70%	66.70%
Histopathology	100	100	100%	100%
Urea Breath Test	96.20%	91.20%	94.70%	93.90%
Stool Antigen Test	92.30%	91.20%	94.40%	86.10%

Triple therapy was the most commonly prescribed regimen (64.1%). However, due to increasing antibiotic resistance, quadruple therapy was utilized in approximately one-third of patients (Table 18).

**Table 18:** Treatment regimens used for H. pylori eradication in the study population

Regimen	Number of Patients
Triple Therapy	50
Quadruple Therapy	28

The adoption of quadruple therapy as first-line treatment, particularly in regions with considerable antibiotic resistance, is supported by the fact that it received a higher eradication rate (26 patients, 92.9%) as compared to triple therapy (38 patients, 76.0%) (Table 19).

**Table 19:** Eradication success rates by treatment regimen

Regimen	Success Rate (%)	Failure Rate (%)
Triple Therapy	76	24
Quadruple Therapy	92.9	7.1

Adverse effects such as nausea/vomiting and diarrhea were more frequent with quadruple therapy. Despite these adverse effects, the higher eradication rate justifies its use (Table 20).

**Table 20:** Adverse effects reported during H. pylori treatment

Effect	Triple Therapy	Quadruple Therapy
Nausea	10	8
Diarrhea	8	7
Metallic Taste	7	3
Abdominal Pain	5	4

Complications such as GI bleeding and ulcer perforation were significantly more frequent among *H. pylori*-positive individuals, highlighting the bacterium's role in severe ulcer-related outcomes (Table 21).

**Table 21:** Association between H. pylori and peptic ulcer complications

Complication	*H. pylori*-positive	*H. pylori*-negative
GI Bleeding	20	4
Perforation	8	1
Obstruction	6	0

*H. pylori*-positive patients had longer hospital stays and a slightly higher complication rate, emphasizing the significance of early detection and management (Table 22).

**Table 22:** Hospitalization duration and outcome in H. pylori-positive vs. H. pylori-negative patients

Group	Avg. Stay (Days)	Recovery Rate	Complications/Prolonged Stay
*H. pylori*-positive	7.8 ± 3.2	70 (89.7%)	8 (10.3%)
*H. pylori*-negative	5.2 ± 2.4	32 (94.1%)	2 (5.9%)

At the six-month follow-up, ulcer recurrence was observed in 24% (12 patients) of patients who received triple therapy, compared to only 7.1% (two patients) in those who received quadruple therapy. This finding suggests that quadruple therapy not only improves *H. pylori *eradication rates but also decreases the likelihood of ulcer recurrence. The overall recurrence rate among treated patients was 17.9%, emphasizing the need for adherence to treatment and lifestyle modifications to prevent relapse (Table 23).

**Table 23:** Recurrence of ulcers after H. pylori treatment at six-month follow-up

Regimen	Recurrence (%)
Triple Therapy	24
Quadruple Therapy	7.1

## Discussion

Age and gender distribution of the study participants

The age and gender distribution of the study participants revealed that the highest prevalence of *H. pylori *infection was in middle-aged individuals, particularly in the 41-50 years age group. This trend aligns with findings from Zamani et al., who reported that H. pylori prevalence increases with age, with the highest rates observed in aged over 40 years [18]. The higher infection rate in this group may be attributed to prolonged exposure to risk factors, dietary habits, and declining immune function. Gender distribution in the current study showed a slight male predominance, though not statistically significant. This aligns with global epidemiological data showing a slightly higher infection rate in males compared to females, as reported in a systematic review by Hooi et al [19]. However, some regional studies have reported a higher prevalence among females, which may be influenced by cultural and socioeconomic factors, including dietary practices and healthcare-seeking behavior.

Socioeconomic status of the participants

The socioeconomic status of the study participants showed that *H. pylori* infection was more prevalent in individuals from low- and middle-income backgrounds, with a significantly lower prevalence in high-income groups. This finding is consistent with previous studies, which have highlighted that *H. pylori* prevalence is closely linked to poor sanitation, crowded living conditions, and limited access to clean water. Ozbey et al. found that infection rates were significantly higher in populations living in developing countries and lower socioeconomic groups, often due to poor hygiene, contaminated water sources, and food handling practices [20]. Additionally, Ali et al. emphasized the role of overcrowding and lack of sewage systems in the Middle East and North Africa, which contribute to increased transmission rates. These findings underscore the need for targeted public health interventions, particularly in low-income populations, to reduce transmission through improved sanitation and hygiene measures [21].

Clinical symptoms and duration of illness in *H. pylori*-positive and negative patients

Epigastric pain was the most commonly reported symptom in *H. pylori*-positive patients, followed by nausea, bloating, melena, and weight loss. This symptomatology is consistent with previous studies, which have identified epigastric pain as the hallmark symptom of *H. pylori*-related gastroduodenal diseases. Xu et al. noted that bloating and nausea are often due to altered gastric acid secretion and motility dysfunction caused by *H. pylori *colonization [6]. The presence of melena in infected patients suggests a higher risk of gastrointestinal bleeding, which *H. pylori *infection significantly increases the risk of ulcer-related complications. The study further showed that symptoms were more severe and prolonged in *H. pylori*-positive patients, supporting the need for early detection and treatment.

Overall prevalence of *H. pylori* in the study population

The overall prevalence of *H. pylori *in this study was 69.6%, which is significantly higher than the global average prevalence of 44.3%, as reported in a meta-analysis by Zamani et al. [18]. However, it aligns more closely with the reported prevalence in developing countries, where infection rates range from 50.8% to 87.7%, depending on environmental and socioeconomic factors. The higher prevalence in this study may be due to regional dietary habits, sanitation issues, and genetic predisposition among the local population. Studies by Wan et al. [22] on Chinese adults and Ali et al. in the Middle East have similarly reported higher prevalence rates in densely populated regions with limited access to clean water and sanitation [21]. This study's findings reaffirm the need for comprehensive eradication programs, particularly in endemic areas, to reduce the burden of infection and its complications.

Prevalence of *H. pylori *by age group

This study found that *H. pylori* prevalence increased with age, with the highest rates observed in the 31-50 years age group. This trend has been consistently reported in multiple studies, including those by Malfertheiner et al. [10] and Hooi et al. [19], who found that infection rates tend to increase with age due to prolonged exposure and cumulative risk factors. The lower prevalence in younger individuals (12-20 years) may be attributed to improved hygiene practices and early exposure to eradication therapies in recent years. However, in some developing countries, studies have shown higher prevalence rates in children, highlighting the role of early-life exposure, poor hygiene, and intra-familial transmission as key determinants of infection. Longitudinal studies tracking the natural history of infection from childhood to adulthood are needed to better understand age-related changes in *H. pylori *epidemiology.

Association of *H. pylori *with smoking and alcohol consumption

The current study found a higher prevalence of *H. pylori* infection among smokers and alcohol consumers, consistent with previous research highlighting lifestyle factors as potential contributors to infection persistence. Ansari et al. reported that smoking increases gastric inflammation and alters mucosal defense mechanisms, making the stomach more susceptible to *H. pylori *colonization [23]. Additionally, smoking has been shown to reduce the effectiveness of eradication therapy, contributing to higher reinfection rates. Similar findings were reported by Ali et al. [21], who demonstrated a strong association between smoking and *H. pylori*-related gastric diseases in Middle Eastern populations. Alcohol consumption was also more prevalent among infected individuals in the present study, suggesting that alcohol may disrupt the gastric microbiome and facilitate *H. pylori *adherence to the mucosa [22]. However, some studies have reported contradictory results, with no significant association between alcohol use and *H. pylori *prevalence, suggesting that additional factors such as drinking patterns and genetic susceptibility may play a role.

Dietary habits and *H. pylori i*nfection

The study found that *H. pylori *infection was more common in individuals consuming high-salt, spicy, and processed foods, supporting existing evidence on dietary risk factors. A review by Wang et al. highlighted that excessive salt intake damages the gastric mucosa, making it more vulnerable to *H. pylori *colonization and inflammation [24]. Moreover, high-salt diets have been linked to increased virulence of *H. pylori *strains, enhancing their ability to cause gastritis and gastric cancer. Spicy food consumption was also associated with a higher infection rate, in agreement with findings from Xu et al. [6], who noted that capsaicin and other spice compounds can exacerbate gastric irritation, leading to worsened symptoms in infected individuals. Similarly, processed food intake was found to be higher among *H. pylori*-positive individuals, consistent with a study by Ozbey et al. [20], which linked preservatives and low-fiber diets to gut dysbiosis, creating an environment favorable for *H. pylori *growth.

Association of *H. pylori *with NSAID use

The study demonstrated a strong association between NSAID use and *H. pylori *infection, with NSAID users exhibiting a significantly higher infection rate than non-users. This finding is supported by Wang et al. [24], who found that NSAIDs and *H. pylori* act synergistically to increase the risk of gastric mucosal injury and ulcer formation. The mechanism involves NSAID-induced inhibition of prostaglandin synthesis, which weakens mucosal defenses against *H. pylori*-mediated damage. Cheng et al. [25] further reported that patients on long-term NSAID therapy are at a higher risk of ulcer complications, especially if infected with *H. pylori*, reinforcing the need for co-testing and eradication strategies in chronic NSAID users.

Association of *H. pylori *with family history of peptic ulcer disease (PUD)

A significant association was observed between *H. pylori *infection and a positive family history of PUD. This result aligns with findings by Liao et al. [26], who noted that familial aggregation of *H. pylori *infection is common, likely due to genetic susceptibility and household transmission. The role of intrafamilial spread through shared utensils, close contact, and contaminated drinking water has been highlighted in studies from endemic regions. Additionally, studies by Wang et al. [24] suggest that genetic variations in inflammatory cytokines, such as IL-6 and TNF-α, may predispose individuals to more severe *H. pylori*-related pathology, explaining the hereditary component of ulcer disease.

Association of *H. pylori *with hygiene practices

Hygiene practices, particularly access to clean drinking water and frequent handwashing, showed a strong correlation with *H. pylori* infection in the present study. Individuals who consumed unfiltered water had nearly double the infection rate compared to those using filtered or boiled water. This finding is supported by Ali et al. [21], who reported that contaminated water sources serve as reservoirs for *H. pylori *transmission, particularly in low-income regions. Poor hand hygiene was also identified as a risk factor, consistent with findings from Xu et al. [6], who noted that fecal-oral and oral-oral transmission routes are facilitated by inadequate hygiene practices. These results emphasize the importance of sanitation, water purification, and hygiene education in reducing infection rates [6].

Endoscopic findings in *H. pylori*-positive and negative patients

The endoscopic findings in this study revealed that duodenal ulcers and erosive gastritis were significantly more common among *H. pylori*-positive patients compared to *H. pylori*-negative individuals. These results are consistent with findings from Elshenawi et al. [27], who demonstrated that *H. pylori *infection is strongly associated with endoscopic features such as gastric nodularity, mucosal swelling, and atrophic changes. Nodularity and mucosal swelling have been shown to be highly specific for *H. pylori *infection, as these findings indicate chronic inflammatory changes induced by bacterial colonization. Furthermore, a systematic review by Cardos et al. [28] highlighted that non-magnified endoscopy has moderate accuracy (67%) in detecting *H. pylori *infection, with key visual markers such as a mosaic pattern, red streaks, and atrophy being strongly predictive. Despite these advancements, the study by Elshenawi et al. [27] emphasizes that endoscopic diagnosis alone may not be sufficient to confirm *H. pylori *infection, necessitating the use of confirmatory diagnostic tests.

Classification of ulcers based on the Forrest classification

The Forrest classification of ulcers in this study demonstrated that active bleeding ulcers (Forrest IA and IB) were more commonly observed in *H. pylori*-positive patients compared to *H. pylori*-negative individuals. This finding aligns with previous studies showing that *H. pylori* infection increases the risk of ulcer hemorrhage due to its role in impairing mucosal healing and increasing local inflammation. A study by Ansari et al. [23] highlighted that chronic *H. pylori*-induced inflammation weakens the gastric mucosal barrier, predisposing patients to severe ulceration and hemorrhage. Moreover, Cheng et al. [25] in their ACG guidelines reported that Forrest IA ulcers (active spurting hemorrhage) have a strong association with *H. pylori *infection, reinforcing the need for aggressive eradication therapy in high-risk patients. These findings suggest that *H. pylori *eradication should be prioritized in patients presenting with high-risk ulcer characteristics to prevent recurrent bleeding episodes.

Histopathological diagnosis of gastric biopsy samples

Histopathological analysis in this study revealed that chronic gastritis and gastric atrophy were the most common findings in *H. pylori*-positive patients. This is in agreement with Xu et al. [6], who demonstrated that gastric atrophy and chronic active gastritis are hallmarks of *H. pylori *infection, driven by bacterial-induced chronic inflammation. The presence of intestinal metaplasia in 12.8% of *H. pylori*-positive patients in this study is also consistent with findings from Malfertheiner et al. [10], who reported that intestinal metaplasia represents a precancerous stage in the Correa cascade, increasing the risk of gastric adenocarcinoma. Furthermore, studies by Wan et al. [22] emphasize that histopathological findings such as lymphoid follicle formation and neutrophilic infiltration are key indicators of *H. pylori*-induced gastric pathology. Given these findings, early detection and eradication of *H. pylori *are crucial in preventing the progression from chronic gastritis to gastric cancer.

Comparison of diagnostic tests used for *H. pylori d*etection

The present study compared the accuracy of different diagnostic methods for *H. pylori*, with histology exhibiting the highest detection rate (69.6%), followed by the UBT (67.0%), SAT (64.3%), and rapid urease test (62.5%). These results align with findings by Ansari et al. [23], who emphasized that histology remains the gold standard for *H. pylori *diagnosis, particularly in patients with atrophic gastritis or intestinal metaplasia. Additionally, Xu et al. [6] noted that the UBT and SAT offer highly reliable, non-invasive diagnostic options, especially in patients unable to undergo endoscopy. However, a systematic review by Malfertheiner et al. [10] highlighted that SAT performance may be affected by PPI use, leading to false-negative results. This study’s findings underscore the need for choosing diagnostic tests based on patient characteristics and clinical settings, with histology preferred for severe cases and UBT/SAT for non-invasive screening.

Sensitivity and specificity of diagnostic tests for *H. pylori*

In terms of diagnostic accuracy, histology demonstrated 100% sensitivity and specificity, while UBT and SAT showed sensitivity rates of 96.2% and 92.3%, respectively. These findings are supported by Cheng et al. [25], who reported that histology offers the highest diagnostic accuracy, particularly in patients with gastric mucosal abnormalities. However, they also highlighted that UBT remains the most reliable non-invasive test, with a sensitivity of 95% in patients not taking PPIs. A study by Wang et al. [24] further supports these findings, emphasizing that false-negative results in RUT and SAT can occur due to antibiotic or PPI use. This study reinforces the importance of test selection based on patient history and clinical presentation, with histology favored in high-risk patients and non-invasive tests preferred for routine screening.

Recurrence of ulcers after *H. pylori t*reatment at six-month follow-up

The present study found an overall ulcer recurrence rate of 17.9% at six months, with significantly higher recurrence in patients who received triple therapy (24.0%) compared to quadruple therapy (7.1%). This difference in recurrence rates aligns with findings from Hu et al. [29], who conducted a global meta-analysis and reported that *H. pylori* recurrence rates are inversely related to the human development index (HDI), with higher recurrence in developing countries due to poor sanitation and reinfection. Their study estimated an annual recurrence rate of 4.3% globally, but rates were significantly higher in regions with inadequate healthcare infrastructure.

Another important finding in this study was that quadruple therapy led to significantly lower recurrence rates, supporting its role as a preferred first-line treatment in areas with high antibiotic resistance. Similar results were observed by Panigrahi et al. [30], who found that quadruple therapy reduced the risk of recurrence due to its ability to overcome metronidazole resistance. Their randomized controlled trial demonstrated that triple therapy had an eradication failure rate of 7%, leading to a higher chance of persistent infection and ulcer relapse [30].

Factors influencing ulcer recurrence and reinfection

The findings of this study suggest that reinfection and recrudescence of *H. pylori *are major contributors to ulcer recurrence, particularly in low-income and high-prevalence regions. Xue et al. [31] conducted a large-scale follow-up study and identified key risk factors associated with *H. pylori *recurrence, including poor hygiene, close contact with infected individuals, and hospitalization. They reported an annual recurrence rate of 1.75% in urban populations but noted that PUD was an independent risk factor for reinfection.

Similarly, Xue et al. [31] found that low-income status, poor sanitation in dining places, and prior invasive diagnostic procedures significantly increased the risk of reinfection. Their findings emphasize the need for improving hygiene practices and ensuring proper sterilization in healthcare settings to prevent nosocomial transmission [31].

Impact of delayed eradication on ulcer recurrence and complications

Another crucial factor influencing ulcer recurrence is the timing of *H. pylori *eradication therapy. Sverdén et al. [32] conducted a population-based cohort study and found that delays in starting eradication therapy significantly increased the risk of recurrent ulcers and ulcer-related complications. Patients who received eradication therapy more than 60 days after ulcer diagnosis had nearly a fourfold higher risk of recurrence compared to those treated within seven days. Their study further highlighted that delayed treatment also increased the likelihood of gastric cancer, reinforcing the importance of early eradication to prevent long-term complications.

Alternative treatment strategies for reducing recurrence

Given the increasing resistance to conventional triple therapy, newer treatment strategies have been explored to improve eradication rates and prevent recurrence. One promising approach is the use of Vonoprazan-based therapy, a potassium-competitive acid blocker (P-CAB) that provides stronger and longer-lasting acid suppression compared to PPIs. A study by Smith et al. [33] demonstrated that vonoprazan-based therapy significantly improves *H. pylori *eradication rates, particularly in resistant strains, and may offer a more effective alternative for reducing ulcer recurrence [33].

Another alternative strategy involves sequential and concomitant therapies, which have shown comparable eradication rates to quadruple therapy. A randomized trial by Hooi et al. [19] compared sequential therapy (ST) and concomitant therapy (CT) in patients with perforated duodenal ulcers and found that both regimens had similar eradication rates and ulcer recurrence rates. However, ST was more cost-effective, making it a viable option for resource-limited settings.

The findings of this study highlight that quadruple therapy is superior to triple therapy in reducing ulcer recurrence, particularly in regions with high antibiotic resistance. However, recurrence remains a persistent challenge due to reinfection, recrudescence, poor hygiene, and delays in eradication therapy. Alternative treatment strategies, such as vonoprazan-based therapy and ST, show promise in improving eradication success rates and reducing long-term ulcer relapse. These findings reinforce the importance of early eradication, personalized treatment approaches, and public health interventions to minimize *H. pylori *reinfection and ulcer recurrence.

Limitations

This study is subject to several limitations. Firstly, its cross-sectional design precludes the establishment of causal relationships between *H. pylori *infection and associated risk factors such as NSAID use, dietary patterns, and hygiene practices. Additionally, as the research was conducted at a single tertiary care hospital in Chennai, the results may not be generalizable to other regions with different population characteristics and healthcare infrastructures.

Several variables - including smoking, alcohol consumption, diet, and hygiene practices - were obtained through self-reported questionnaires, which are vulnerable to recall bias and social desirability bias. Moreover, although treatment outcomes and ulcer recurrence were evaluated, the follow-up period was limited to six months, which may not adequately reflect long-term recurrence or delayed complications.

Finally, the study did not include antimicrobial susceptibility testing, despite the acknowledged challenge of rising antibiotic resistance. This omission limits the ability to link treatment outcomes with specific resistance profiles, which are essential for developing region-specific therapeutic strategies.

## Conclusions

A Chennai-based study reveals a high *H. pylori *prevalence (69.6%) in duodenal ulcer patients, exceeding global averages. It is most common in middle-aged individuals and linked to low socioeconomic status, poor hygiene, diet, NSAID use, and family history. Infected patients showed more severe symptoms, higher bleeding rates (25.6%), and longer hospital stays.

Quadruple therapy (92.9% eradication) proved superior to triple therapy (76.0%), despite more side effects, significantly reducing ulcer recurrence. The study highlights the need for early diagnosis, treatment, improved public health, and antibiotic susceptibility testing due to recurrence risks and resistance.
